# The Type 2 Diabetes Susceptibility PROX1 Gene Variants Are Associated with Postprandial Plasma Metabolites Profile in Non-Diabetic Men

**DOI:** 10.3390/nu11040882

**Published:** 2019-04-19

**Authors:** Edyta Adamska-Patruno, Joanna Godzien, Michal Ciborowski, Paulina Samczuk, Witold Bauer, Katarzyna Siewko, Maria Gorska, Coral Barbas, Adam Kretowski

**Affiliations:** 1 Clinical Research Centre, Medical University of Bialystok, 15-089 Bialystok, Poland; joannagodzien@gmail.com (J.G.); michal.ciborowski@umb.edu.pl (M.C.); paulina.samczuk@gmail.com (P.S.); witold.bauer@umb.edu.pl (W.B.); adamkretowski@wp.pl (A.K.); 2 Department of Endocrinology, Diabetology and Internal Medicine, Medical University of Bialystok, 15-089 Bialystok, Poland; katarzynasiewko@o2.pl (K.S.); mgorska25@wp.pl (M.G.); 3 Center for Metabolomics and Bioanalysis (CEMBIO), Universidad CEU San Pablo, 28003 Madrid, Spain; cbarbas@ceu.es

**Keywords:** nutrigenetics, nutrimetabolomics, high-carbohydrate meal, normo-carbohydrate meal, postprandial metabolic fingerprinting, ultra-high performance liquid chromatography, PROX1 gene, type 2 diabetes mellitus risk

## Abstract

The prospero homeobox 1 (PROX1) gene may show pleiotropic effects on metabolism. We evaluated postprandial metabolic alterations dependently on the rs340874 genotypes, and 28 non-diabetic men were divided into two groups: high-risk (HR)-genotype (CC-genotype carriers, *n* = 12, 35.3 ± 9.5 years old) and low-risk (LR)-genotype (allele T carriers, *n* = 16, 36.3 ± 7.0 years old). Subjects participated in two meal-challenge-tests with high-carbohydrate (HC, carbohydrates 89%) and normo-carbohydrate (NC, carbohydrates 45%) meal intake. Fasting and 30, 60, 120, and 180 min after meal intake plasma samples were fingerprinted by liquid chromatography quadrupole time-of-flight mass spectrometry (LC-QTOF-MS). In HR-genotype men, the area under the curve (AUC) of acetylcarnitine levels was higher after the HC-meal [+92%, variable importance in the projection (VIP) = 2.88] and the NC-meal (+55%, VIP = 2.00) intake. After the NC-meal, the HR-risk genotype carriers presented lower AUCs of oxidized fatty acids (−81–66%, VIP = 1.43–3.16) and higher linoleic acid (+80%, VIP = 2.29), while after the HC-meal, they presented lower AUCs of ornithine (−45%, VIP = 1.83), sphingosine (−48%, VIP = 2.78), linoleamide (−45%, VIP = 1.51), and several lysophospholipids (−40–56%, VIP = 1.72–2.16). Moreover, lower AUC (−59%, VIP = 2.43) of taurocholate after the HC-meal and higher (+70%, VIP = 1.42) glycodeoxycholate levels after the NC-meal were observed. Our results revealed differences in postprandial metabolites from inflammatory and oxidative stress pathways, bile acids signaling, and lipid metabolism in PROX1 HR-genotype men. Further investigations of diet–genes interactions by which PROX1 may promote T2DM development are needed.

## 1. Introduction

Type 2 diabetes mellitus (T2DM) is a major public health issue affecting 415 million people worldwide in 2015 [1], and it is expected that it will affect over 439 million people by 2030 [2] and 642 million by 2040 [1]. The T2DM is characterized by impaired β-cell function and insulin resistance, which leads to chronic hyperglycemia [3].

The Genome-Wide Association Studies (GWAS) and other, different scale meta-analyses and studies have indicated that the rs340874 single nucleotide polymorphism (SNP) in the prospero homeobox 1 (PROX1) gene is a strong genetic susceptibility factor for T2DM [4,5,6]. It has been shown that allele C of rs340874 is associated with reduced insulin sensitivity, β-cell function, insulin secretion, and fasting glucose levels [7,8,9]. PROX1 encodes a key transcription factor (TF), which is involved in the development of tissues such as pancreas [10]. It has been also suggested that reduced expression of PROX1 results in altered β-cell insulin secretion and thereby confers the T2DM susceptibility [5]. In one of our previous studies [11], we noted that carriers of the rs340874 PROX1 CC genotype presented higher free fatty acids levels after a high-fat meal intake and lower glucose utilization after a high-carbohydrate meal intake. Moreover, in subjects carrying the CC genotype, we found higher visceral fat accumulation despite lower daily food consumption, which indicates that another potential pathway may be involved in T2DM development in people at high genetic risk. Taken together, the studies show that PROX1 variants may have a pleiotropic effect on metabolism; however, the link between PROX1 and T2DM has not been established to date. Detailed characterization of PROX1 genetic variability can help to elucidate the role of PROX1 gene variations in T2DM development and to explore its potential pathways.

We hypothesize that one of the pathways involved in the T2DM development in subjects with the PROX1 rs340874 CC genotype may be a lipid metabolism path, and its further oxidative stress consequences can be modulated by different diets with varying macronutrients content. In our previous studies, we found that some subtle metabolism alterations are detectable only postprandially, and since most of the daytime people spend in the post absorptive state, the postprandial metabolism may play a crucial role in metabolic disorders development and/or progression [12]. We observed in our studied group that the differences in the postprandial metabolic response depend on many factors such as actual nutritional status [13,14,15] but also depend on genotype [11,16,17].

Studies carried out so far—as well as our own observations—indicate that the mechanisms by which the PROX1 gene affects the susceptibility to T2DM seem to be more complex. Therefore, for further investigation, we used the metabolomics approach. We used a liquid chromatography quadrupole time-of-flight mass spectrometry (LC-QTOF-MS) to evaluate postprandial changes in serum metabolites during the high-carbohydrate (HC) and normo-carbohydrate (NC) meal-challenge-tests in non-diabetic men dependent on the PROX1 rs340874 genotypes.

## 2. Materials and Methods

### 2.1. Subjects

The volunteers for our meal test study [14,15,16,17] were recruited from the 1000PLUS cohort study of Polish origin Caucasian population [11,18,19]. This trial was registered at www.clinicaltrials.gov as NCT03792685. Only males were enrolled into the meal-challenge-tests because of the possible sexual dimorphism of investigated factors [20]. The study participants (*n* = 28) were divided into 2 groups dependent on the PROX1 rs340874 genotypes: the homozygous carriers of high-risk (HR) allele C (CC genotype, *n* = 12) and carriers of low-risk (LR) allele T (both CT and TT genotypes, *n* = 16). None of the participants suffered from T2DM, prediabetes, or other disorders, nor did they report any treatments that might affect the tests results. Subjects who followed any special diet or dietary patterns (vegetarian, high-fat, etc.) were excluded from the experiment.

### 2.2. Ethics

The study procedures were conducted in accordance with all of the ethical standards of human experimentation and with the Declaration of Helsinki. The study protocol was approved by the local Ethics Committee (Medical University of Bialystok, Poland, R-I-002/35/2009), and before any study procedures, all of the participants signed informed consent.

### 2.3. Study Procedures

At the screening visit, the demographic data and anthropometric measurements, body weight, body composition analysis, oral glucose tolerance test (OGGT), and blood collections for biochemical and genotype analyses were performed as described previously [11,18]. Only men were enrolled into the meal-challenge-tests. Participants were instructed to maintain their regular lifestyle throughout the study and to avoid alcohol, coffee, and excessive physical exercise at least on the day before each test. The meal-challenge-test visits were conducted as described previously [14,15,16,17,21]. Briefly, the volunteers participated in two meal-challenge-tests visits in crossover design at an interval of 2–3 weeks. After an overnight fast, the participants arrived at the laboratory, and after fasting blood collection, they received (in random order) a standardized HC-meal (300 mL, Nutridrink Juice Style, Fat Free, Nutricia, Poland), which provided 450 kcal (89% of energy from carbohydrate, 11% from protein, and 0% from fat), or NC-meal (360mL, Cubitan, Nutricia, Poland), providing 450 kcal (45% of energy from carbohydrate, 30% from protein, and 25% from fat). During the whole experiment, men stayed in bed in a quiet room with thermoneutral conditions (22–25 °C). The metabolomics analyses were performed on plasma samples from the blood collected at fasting and at 30, 60, 120, and 180 min after meal intake.

### 2.4. Metabolomics Analysis

The metabolomics analysis is described in detail in the Supplementary Materials. Briefly, metabolic fingerprinting was performed on an HPLC system (1290 Infinity, Agilent Technologies, Santa Clara, CA, USA) coupled to an iFunnel Q-TOF (6550, Agilent Technologies, Santa Clara, CA, USA) mass spectrometer. Plasma samples were prepared and analyzed (in positive and negative ion modes) following previously described protocols and methods [22].

Data treatment included cleaning of background noise and unrelated ions through molecular feature extraction (MFE) tool in Mass Hunter Qualitative Analysis Software (B.06.00, Agilent, Santa Clara, CA, USA). Mass Profiler Professional (B.12.61, Agilent Technologies, Santa Clara, CA, USA) software was used to perform quality assurance (QA) procedure and data filtration. QA procedure covered a selection of metabolic features with good repeatability. To achieve the features detected in >80% in quality control (QC) samples and with RSD <30% (as calculated for the QC samples) in NC- and/or HC-meals, the dataset was kept for further data treatment. Additional data filtering was performed considering biological samples. Data were divided into ten sets with five time-points: 0, 30, 60, 120, and 180 min in two meal challenge groups. Metabolic features present in ≥80% of samples in at least one of these datasets were accepted. Moreover, a dedicated filtering for each comparison was performed—metabolic features present in a minimum of 80% of samples from one group were forwarded for statistical analysis. Detailed information about analytical conditions is available in the Supplementary Materials.

### 2.5. Calculations

Based on the relation between time points and the signal intensity of each metabolite, the areas under the curve (AUCs) were calculated using a trapezoid rule in R software environment (version 3.4.3, https://www.R-project.org/). The Homeostatic Model Assessment of Insulin Resistance (HOMA-IR) was calculated using the standard formula [23]:

HOMA-IR = fasting plasma glucose concentration (mmol/L)] × fasting insulin concentration (µU/mL)]/22.5

The Homeostatic Model Assessment of β-cell function (HOMA-B) was calculated using the following formula [23]:

HOMA-B = 20 × fasting insulin (µU/mL)/fasting glucose (mmol/L) − 3.5

### 2.6. Statistical Analysis

Statistical analysis was performed on each metabolite’s mean AUCs within different strata. Patients with the HR-genotype (CC genotype) were compared to patients with the LR-genotype carrying the protective allele T (CT/TT genotypes) in rs340874 of the PROX1 gene. NC- and HC-meal groups were analyzed independently. Selection of statistically significant metabolites was performed implementing both, uni-, and multivariate analyses. For each significant metabolite, *p*-value was calculated in Matlab (MathWorks Inc.). The Shapiro-Wilk test was used for normality testing and then, dependent on the data distribution, the *t*-test or the Mann-Whitney test were performed. Partial least square discriminant analysis (PLS-DA) models were computed using the SIMCA software (Umetrics). Based on PLS-DA models, volcano plots were created plotting variable importance in the projection (VIP) against corrected *p*-values [*p*(corr), loading values scaled as correlation coefficients values]. Variables with VIP >1.0 and absolute *p*(corr) >0.4 were considered significant.

### 2.7. Identification

Statistically significant metabolites were annotated by matching the spectral data from public databases (HMDB, METLIN, and LIPIDMAPS) with spectral data obtained through MSMS (tandem MS—mass spectrometry) analysis for metabolites present in plasma samples. Detailed information about identified metabolites is included in the Supplementary Materials (Table S1).

## 3. Results

### 3.1. Baseline Characteristics

The baseline characteristic of the studied population is presented in Table 1. The studied genotypes groups were well matched without any between-group differences in age, anthropometric measurements, body mass index (BMI), body fat and fat free mass content, fasting glucose and insulin concentrations, HOMA-IR, HOMA-B, and glycated hemoglobin (HbA1c).

### 3.2. Genotype Effects on Metabolites Profiles

Samples were divided into two groups according to the type of meal taken, NC- and HC-meals, and then analyzed in independent analytical batches in both polarity modes. This resulted in four datasets, which were aligned according to their polarity: ESI+ for both meals and ESI- for both meals. After application of the QA procedure, there were 1717 metabolic features in ESI+ and 848 in ESI- past QA procedure from both data sets (NC- and HC-meals). Principal component analysis (PCA) was implemented to visualize the results of the QA procedure. For each analytical sequence, the QC samples clustered tightly (Figure S1), which indicated the system’s stability and therefore the good quality of the data.

Final datasets contained only features presented in ≥80% of the samples in at least one of the two studied groups (CC versus CT/TT). It resulted in 1494 and 843 features for the NC- and HC-meals in ESI+ mode and the NC- and HC-meal in ESI- mode, respectively.

**Figure 1:**
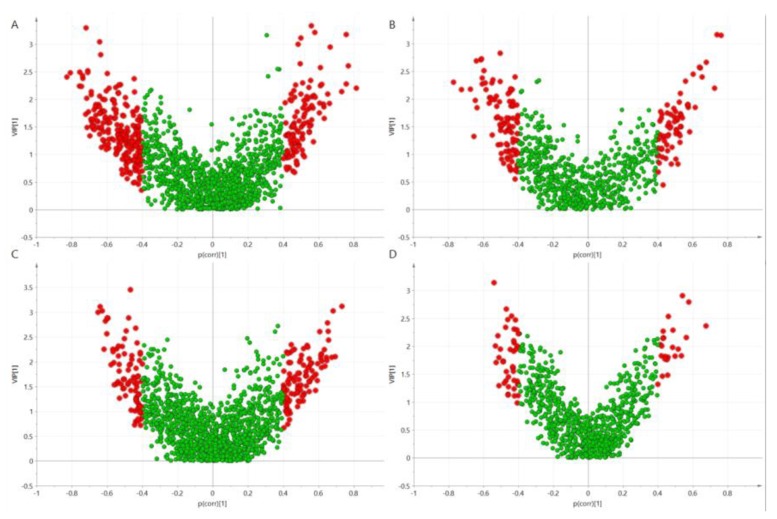
Volcano plots build on the Partial least square discriminant analysis (PLS-DA) models computed based on the area under the curves (AUCs) of plasma metabolites after norma-carbohydrate (NC)-meal for ESI+ (**A**) and ESI- (**B**) and high carbohydrate (HC)-meal for ESI+ (**C**) and ESI- (**D**). Red color marks metabolic features significantly differenting HR-genotype (CC) and LR-genotype (CT/TT) carriers.

**Figure 2:**
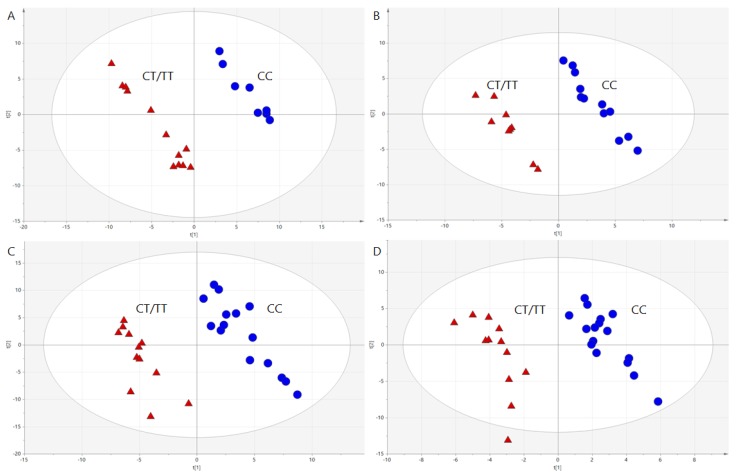
PLS-DA models computed based on AUCs of plasma metabolites illustrating clear separation between men carrying CC (blue dots) and CT/TT (red triangles) genotypes after NC-meal for ESI+ (**A**) and ESI- (**B**), and HC-meal for ESI+ (**C**) and ESI- (**D**). The parameters of the models: R2 = 0.989, Q2 = 0.279 for NC-meal for ESI+; R2 = 0.989, Q2 = 0.433 for NC-meal for ESI-; R2 = 0.947, Q2 = 0.490 for HC-meal for ESI+; R2 = 0.943, Q2 = 0.109 for HC-meal for ESI-. R2 = explained variance, Q = predictive capability of the model.

To select discriminating metabolites, volcano plots (Figure 1) were built based on PLS-DA models (Figure 2). Studied genotypes did not differ significantly in fasting metabolite profiles, however, metabolic profiles changed and differed between genotypes after the meal intake.

Metabolites discriminating studied genotypes after both meals are presented in Table 2. For all of the identified metabolites, the calculated error of the measured mass in comparison to the theoretical monoisotopic mass was ≤4 ppm.

**Figure 3:**
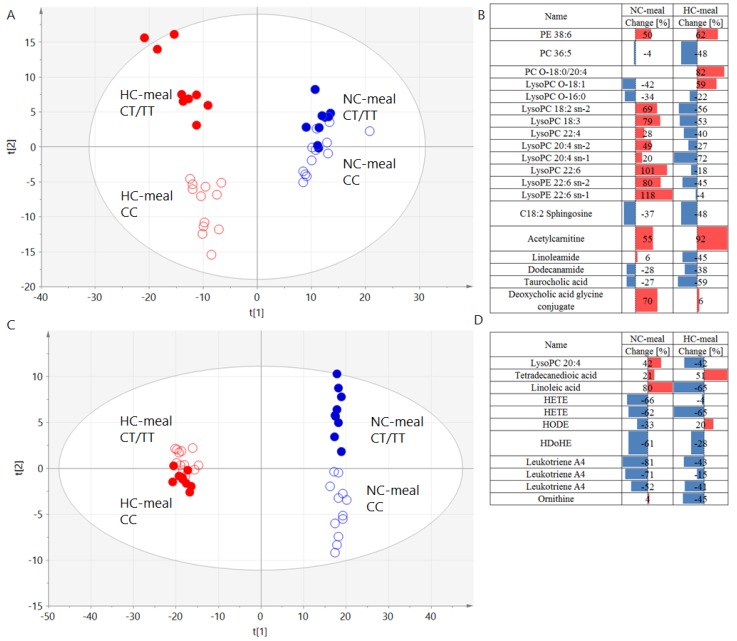
PLS-DA models computed based on AUCs of plasma metabolites illustrating clear separation between men carrying CC (empty dots) and CT/TT (full dots) genotypes after NC- (blue color) and HC-meal (red color) for ESI+ (panel **A**) and ESI- (panel **C**) with the summary of the differentiating signals and their change in ESI+ (panel **B**) and ESI- (panel **D**). The parameters of the models: R2 = 0.581, Q2 = 0.318 for ESI+; R2 = 0.594, Q2 = 0.0.264 for ESI-. R2 = explained variance, Q = predictive capability of the model.

Global overview of all the samples revealed that there was a difference in the metabolic response to the meal between men carrying CC and CT/TT genotypes (Figure 3). Interestingly, the direction of these changes was different between the two polarity modes applied and therefore was related to the type of molecules measured. In ESI+ lipids, different lipid classes were changing (in majority of the cases) opposite to the way they were changing between men with different genotypes. In ESI-, most of the molecules exhibited the same direction of change but with differences in the magnitude of the change.

We did not observe any crucial differences between studied genotypes in the fasting plasma metabolites profile, as mentioned above. Postprandially, we noted that the AUCs of the postprandial very long chain unsaturated PC36:5 levels were lower after NC-meals, while the AUCs of PE38:6 levels were significantly higher after both meal intakes in the HR-group. The HR-genotype carriers presented lower AUCs after the HC-meal and higher AUCs after the NC-meal for postprandial levels of polyunsaturated LysoPC and LysoPE with 18-22-carbon chain length. Conversely, the AUCs of the monounsaturated and the saturated LysoPCs postprandial levels (18 and 16 carbons in length) after the NC-meal intake in the HR-genotype men were lower compared to the LR-genotype carriers. We also noted higher AUCs of postprandial linoleic acid (LA) levels, lower AUCs of hydroxyeicosatetraenoic acids (HETE), and hydroxyoctadecenoic acid (HODE) levels after NC-meal intake, and after both meals, we noted lower AUCs of postprandial hydroxydocosahexaenoic acid (HDoHe) levels. After the HC-meal intake in HR-genotype men, we observed higher AUCs of postprandial tetradecanedioic acid. The HR-genotype men presented lower AUCs of postprandial leukotriene A4 (LTA4) and sphingosines levels and higher AUCs of postprandial acylcarnitines levels after both meal intakes. Lower AUCs of postprandial linoleamide levels after the HC-meal, and for dodecanamide levels, after both meal intakes, were observed. The AUCs of postprandial taurocholic acid levels were lower after the HC-meal intake, while the AUCs of deoxycholic acid glycine conjugated were higher after the NC-meal intake. Moreover, we noticed lower AUCs of postprandial ornithine levels after the HC-meal intake in the HR-genotype men.

## 4. Discussion

We evaluated the metabolomics analyses at fasting and postprandial states to explore the impacts of the rs340874 SNP in the PROX1 gene on the human metabolism. At the fasting state, we did not observe any crucial differences in metabolites levels between studied genotypes, but the meal-challenge-tests uncovered several postprandial alterations. We noted some differences in the postprandial phospholipid levels. Altered PCs and LPCs plasma profiles were associated with T2DM [24]. LPCs were reduced in subjects with diabetes [25] and with insulin resistance [26]. Our participants were free from T2DM and prediabetes states, and HR-genotype carriers did not differ in insulin sensitivity from the men carrying the LR-genotype. The changes in postprandial LPCs levels, typical for insulin resistance and T2DM, were induced mostly by the HC-meal consumption, but after the NC-meal intake, the HR-genotype carriers presented significantly higher AUCs of some postprandial LPCs levels. Yea K. et al. [27] showed that LPCs could stimulate glucose uptake via an insulin-independent mechanism. This is consistent with the results from our previous study, which showed that genotype CC carriers who presented lower AUC of postprandial LPCs after a HC-meal intake in this study also presented lower postprandial glucose utilization and higher blood glucose concentrations after the HC-meal intake in our previous experiment [11].

In HR-genotype men after the NC-meal intake, we also noticed higher AUCs of postprandial LA and lower AUCs of postprandial levels of long-chain fatty acids (LCFA) esters derived from arachidonic acid (AA), HETE, and from docosahexaenoic acid (DHA), the HDoHe, after both meal intakes. Some of the oxidized fatty acids had a biological activity and could signal through their own receptors to evoke a variety of physiological changes. It has been shown that reduced glucose-induced 20-HETE formations and release contribute to inefficient glucose-stimulated insulin secretion in islets isolated from T2DM humans and mice [28]. Moreover, after NC-meal intake, we noted the HR-genotype men had lower AUCs of postprandial levels of another hydroxy fatty acid—HODE. The HODEs are likely to be exogenous activators and natural ligands for the nuclear receptor peroxisome proliferator-activated receptors α (PPARα) [29] and PPARγ [30]. The PPARα is an important mediator of metabolic response to nutritional factors since it is involved in fasting and postprandial lipid metabolism regulation, as well as in the mechanisms associated with body energy production. However, it also modulates the transcription of genes involved in pathways of inflammatory responses [31]. PPARγ receptors play a key role in the insulin sensitization, adipocyte differentiation, and adipose tissue lipid metabolism dependent on nutritional state—the highest postprandial expression and activation leads to the upregulation of genes that mediate fatty acids trapping and uptake [32,33]. Adipose PPARγ protects nonadipose tissue against lipid overload [34], and the use of PPARγ agonists has been shown to cause a shift of fat distribution from visceral to subcutaneous adipose depots, which is associated with improvements in hepatic and peripheral tissue insulin sensitivity [35]. It has been found that the activation of PPARα and PPARγ attenuates total free fatty acid and triglyceride accumulation, which may reduce the risk of obesity, diabetes, and atherosclerosis [36]. Therefore, as was noted in our study, lower postprandial HODE levels (which are natural ligands for the PPARα and PPARγ receptors) in the HR-genotype men may have disadvantageous effects. Results from our larger cohort population have indicated that the PROX1 HR-genotype carriers present significantly higher visceral fat accumulation [11].

The HR-genotype men also presented higher AUCs of postprandial tetradecanedioic acid levels after the HC meal intake, which may suggest an altered peroxisomal beta-oxidation since the oxidation of tetradecanedioic acid has been found to be reduced by more than 75% in peroxisome deficient hepatocytes [37].

Our metabolomics analysis showed lower AUCs of postprandial leukotriene A4 (LTA4) plasma levels in the HR-genotype carriers after both meal intakes. The LTA4 could be further metabolized to form LTB4, which plays an important role in metabolic disruptions [38]. Therefore, as was noted in our study, lower LTA4 may be a beneficial symptom or may indicate the higher conversion into LTB4. It has been already shown that PROX1 is associated with defects affecting lymphatic vascular structure and function, which may lead to lymphedema and imbalanced eicosanoid metabolism [39,40].

After both meal intakes, the HR-risk genotype carriers presented higher AUCs of postprandial acylcarnitine levels. Increased plasma acylcarnitines levels have been reported in insulin-resistant and T2DM subjects as products of incomplete or inefficient β-oxidation, and tissue accumulation of acylcarnitine molecules may lead to activation of proinflammatory pathways, which are implicated in insulin resistance and T2DM development [41].

Furthermore, in the HR-genotype men, we also noticed lower AUCs of postprandial levels of two fatty acid amides (FAAs)—dodecanamide after both meals and linoleamide after the HC-meal intake. The FAAs may play roles as endogenous brain cannabinoid receptor ligands and may be involved in T2DM pathogenesis [42]. Moreover, linoleamide inhibits phospholipase A2 (PLA2) [43], the suppression of which protects against diet-induced obesity and diabetes, and PLA2-deficient mice presented increased postprandial hepatic fatty acid oxidation (FAO) [44].

We also observed lower AUCs of postprandial sphingosine levels after both meals in people in the HR-genotype group. Sphingosine is a breakdown product of ceramide degradation, and free sphingosine can be trapped for ceramide regeneration or for sphingosine-1-phosphate (S1P) formation, both of which have been associated with obesity, insulin resistance, and T2DM [45,46,47]. Lower plasma sphingosine levels observed in our study could have been a result of decreased release to the circulation (from ceramide degradation, etc.,) or increased rate of its intracellular acylation or phosphorylation, and both possibilities may have had a negative effect—the increase of cellular ceramide and S1P levels. Further studies are needed to elucidate the possible associations between the PROX1 HR-genotype and the sphingomyelin pathway.

The very interesting finding of our study is that the PROX1 gene may be involved in the postprandial bile acids signaling. After the HC-meal intake in the HR-genotype men, we noticed lower AUCs of postprandial taurocholic acid and higher AUCs of postprandial deoxycholic acid glycine conjugate levels after the NC-meal. It has been found that PROX1 suppress the transcription of the CYP7A1 gene, which codes the key enzyme of bile acid synthesis and may negatively modulate the bile acids synthesis [48]. Bile acids are metabolic regulating factors that act as signaling molecules through receptor-independent and receptor-dependent pathways, including nuclear farnesoid X receptor (FXR) and the membrane Takeda G protein-coupled receptor (TGR), which are implicated in the regulation of glucose, lipid, and energy metabolism. Dysregulation of these pathways may contribute to the metabolic disturbances and T2DM development [49,50,51]. The mechanisms by which bile acids are involved in glucose homeostasis regulation remain undefined. Many different subtypes of bile acids differ widely in their chemical composition as well as in their overall impact on health. Zaborska et al. [52] found that dietary supplementation of deoxycholic acid impairs whole body glucose regulation in mice by disrupting hepatic endoplasmic reticulum homeostasis, and in our experiment, the HR-genotype carriers presented higher AUCs of postprandial deoxycholic acid glycine conjugate levels after the NC-meal intake.

The carriers of the PROX1 HR-genotype also presented lower AUCs of postprandial ornithine levels after HC-meal intake. Increased ornithine levels as a product of arginine catabolism are associated with hyperglycemia and can be involved in the pathogenesis of T2DM [53,54], but lower ornithine levels may be an effect of an increased plasma arginase activity, which is increased in diabetic subjects and may be associated with vascular complications [54].

The PROX1 gene has been shown, thus far, to confer the susceptibility to T2DM mostly by its associations with fasting and glucose-stimulated insulin secretion [5] as well as fasting [9] and OGTT 2-h glucose levels [55]. Our study revealed other postprandial disruptions in the high-risk PROX1 genotype carriers, which may be a part of potential type 2 diabetes disease pathways. The summarizing of all metabolic alterations mentioned above is presented in Figure S2. Most of the alterations we found were observed after HC-meal intake, but the differences between studied genotypes in postprandial levels of molecules involved in pathways of inflammatory and oxidative stress responses were more pronounced after NC-meal intake. Oxidative stress impacts progressive disorders and is linked to metabolic disorders such as T2DM, since the activation of stress pathways plays a key role in the development of the insulin resistance and impaired insulin secretion [56]. We observed the differences between genotypes mostly by the NC-meal intake. This was perhaps due to the fact that the HC-meals induced a strong inflammatory response by themselves [57], and therefore the impact of the genetic risk could have been blurred—especially with a small sample size, which was a major limitation of our study. The main reasons for the small study sample were that a non-targeted LC-MS-based metabolomics approach could be performed in a limited set of samples, and, moreover, the presented study is a part of our larger project with very long and laborious protocol and procedures, which limited the number of volunteers participating.

## 5. Conclusions

In conclusion, our results showed an altered postprandial metabolite profile in the PROX1 HR-genotype carriers. Our observations indicate that one of the pathways involved in the T2DM development in subjects with the PROX1 CC genotype may be postprandial alterations, but further functional studies are required to extrapolate implications from our findings for the biochemical pathways associated with PROX1 SNPs and T2DM development. It may allow identifying the pathways and factors that interact with some dietary nutrients, leading to particular metabolic responses that are associated with the development of metabolic diseases.

## Figures and Tables

**Table 1:** The baseline characteristic of studied population by the rs340874 PROX1 genotypes.

	CC Genotype	CT/TT	*p*-Value *
Age (years)	35.3 ± 9.5	36.3 ± 7.0	0.75
Weight (kg)	93.6 ± 24.5	89.1 ± 16.1	0.95
Body mass index (BMI) (kg/m^2^)	29.1 ± 8.1	27.3 ± 4.2	0.74
Body fat content (%)	23.8 ± 10.1	23.2 ± 7.8	0.87
Fat free mass (%)	69.6 ± 11.0	67.6 ± 8.3	0.60
Waist (cm)	99.6 ± 21.1	95.7 ± 13.6	0.77
Hip (cm)	104.3 ± 14.8	99.6 ± 8.7	0.76
WHR	0.9 ± 0.1	1.0 ± 0.1	0.81
Fasting glucose concentration (mg/dl)	86.2 ± 8.0	86.7 ± 6.4	0.85
Fasting insulin concentration (IU/mL)	10.4 ± 9.1	8.9 ± 5.4	0.84
HOMA-IR	2.2 ± 2.0	1.9 ± 1.3	0.81
HOMA-B	188.2 ± 163.3	143.7 ± 88.9	0.78
HbA1c	5.2 ± 0.5	5.2 ± 0.2	0.90

* For quantitative variables with normal distribution, the parametric *t*-test was used; for the other variables, the non-parametric Mann–Whitney test was applied. The data are represented as the mean ± STD, and *p*-values < 0.05 were considered significant. * HOMA-IR = Homeostatic Model Assessment of Insulin Resistance; HOMA-B = Homeostatic Model Assessment of β-cell function; HbA1c = glycated hemoglobin; CC = high risk genotype; CT/TT = low risk genotype; WHR = waist-hip ratio.

**Table 2:** The percentage differences in AUCs of postprandial plasma metabolite levels after NC-meal and HC-meal intake in the PROX1 high-risk-genotype (CC) men compared to the low-risk genotype carriers (CT/TT).

Name	Molecular Weight, Da	RT, Min	NC-Meal				HC-Meal			
			Change, %	*p*-Value	*p* (corr)	VIP	Change, %	*p*-Value	*p* (corr)	VIP
PE 38:6	763.5152	9.40	50	0.25	0.43	1.38	62	0.18	−0.56	1.98
PC 36:5	779.5465	7.95	−4	0.93	−0.51	1.35	−48	0.19	0.30	1.43
PC O-18:0/20:4	795.6141	10.20			−0.45	1.52			−0.16	1.34
LysoPC O-18:1	507.3689	5.95	−42	0.17	−0.59	2.21	59	0.45	0.27	0.46
LysoPC O-16:0	481.3532	5.80	−34	0.50	−0.64	2.81	−22	0.61	0.34	1.30
LysoPC 18:2 sn-2	519.3325	5.40	69	0.30	0.30	1.07	−56	0.049	0.55	1.90
LysoPC 18:3	517.3168	5.05	79	0.28	0.16	0.71	−53	0.26	0.53	1.72
LysoPC 22:4	571.3638	5.85	28	0.47	0.55	1.51	−40	0.018	0.56	1.96
LysoPC 20:4	543.3325	5.40	42	0.46	0.21	1.01	−42	0.01	0.62	2.16
LysoPC 20:4 sn-2	543.3325	5.35	49	0.07	−0.50	1.43	−27	0.11	0.45	1.76
LysoPC 20:4 sn-1	543.3325	5.35	20	0.74	−0.01	0.07	−72	0.02	0.54	2.91
LysoPC 22:6	567.3325	5.40	101	0.04	0.81	2.21	−18	0.40	0.26	1.11
LysoPE 22:6 sn-2	525.2855	5.35	80	0.23	−0.54	2.35	−45	0.29	−0.34	1.26
LysoPE 22:6 sn-1	525.2855	5.35	118	0.09	−0.56	2.03	−4	0.86	−0.27	1.10
Tetradecanedioic acid	258.1831	4.35	21	0.44	−0.20	0.78	51	0.005	−0.53	2.01
Linoleic acid	280.2402	7.05	80	0.01	−0.59	2.29	−65	0.13	−0.12	0.67
HETE	320.2351	5.70	−66	0.10	0.64	2.56	−4	0.93	0.38	1.69
HETE	320.2351	5.70	−62	0.10	0.57	1.89	−65	0.13	0.43	2.15
HODE	298.2508	5.85	−33	0.09	0.51	1.43	20	0.43	−0.26	1.06
HDoHE	344.2351	5.70	−61	0.11	0.57	1.90	−28	0.53	0.43	2.15
C18:2 Sphingosine	297.2668	5.85	−37	0.25	−0.41	1.60	−48	0.01	0.65	2.78
Leukotriene A4	318.2195	5.45	−81	0.02	0.74	3.16	−43	0.21	0.16	0.79
Leukotriene A4	318.2195	5.45	−71	0.01	0.64	2.58	−15	0.71	0.24	1.14
Leukotriene A4	318.2195	5.45	−52	0.10	0.58	1.40	−41	0.14	0.41	2.03
Acetylcarnitine	203.1158	0.25	55	0.06	0.54	2.00	92	0.002	−0.60	2.88
Linoleamide	279.2562	5.30	6	0.93	−0.01	0.05	−45	0.09	0.53	1.51
Dodecanamide	199.1936	5.20	−28	0.49	−0.81	2.48	−38	0.16	0.48	1.10
Taurocholic acid	515.2917	2.30	−27	0.35	−0.39	0.39	−59	0.07	0.66	2.43
Deoxycholic acid glycine conjugate	449.3141	4.30	70	0.14	0.55	1.42	6	0.75	−0.29	0.91
Ornithine	132.0899	0.25	4	0.83	0.12	0.05	−45	0.047	0.53	1.83

VIP = variable importance in the projection, RT = retention time.

## Supplementary Materials

The following are available online at https://www.mdpi.com/2072-6643/11/4/882/s1, Metabolomics analysis- methods detailed description, Figure S1: The QC of performed analyses- PCA plots with marked QC samples, Figure S2: The summary of metabolic alterations observed after NC- and HC-meal intake, Table S1: Detailed information about compounds identification.
