# Supplementary material for: The Type 2 Diabetes Susceptibility PROX1 Gene Variants Are Associated with Postprandial Plasma Metabolites Profile in Non-Diabetic Men
Source: Nutrients. 2019 Apr 19;11(4):882. doi: 10.3390/nu11040882 (PMC6520869; doi:10.3390/nu11040882)
Supplement: Supplementary file 1 [file nutrients-11-00882-s001.pdf]

Metabolomics analysis- methods detailed description

Chemicals and reagents. Ultrapure water, used to prepare all the aqueous solutions, was obtained "in house" from a Milli-Qplus185 system (Millipore, Billerica, MA, USA). LC-MS-grade methanol and acetonitrile, LC-grade ethanol and analytical grade formic acid were purchased from Fluka Analytical (Sigma-Aldrich Chemie GmbH, Steinheim, Germany). The API-TOF reference mass solution kit (G1969-850001), tuning solutions, ESI-L low concentration tuning mix (G1969-85000) and ESI-TOF Biopolymer Analysis reference masses (G1969-850003) were purchased from Agilent Technologies (Santa Clara, California, USA).

Metabolomics fingerprinting by LC-MS. Plasma samples were prepared by simple protein precipitation and metabolite extraction with three volumes of ice-cold mixture of methanol/ethanol (v/v 1:1) added to one volume of plasma. Samples were vortex-mixed and stored on ice for five minutes. After centrifuging at 16000 × *g* for 10 minutes in 4 °C, supernatant was collected and filtered through a 0.22 μm nylon filter.

Quality control (QC) samples were used to check the stability of the LC-MS system and the reproducibility of the sample treatment. QCs were prepared by pooling aliquots from all investigated plasma samples, and extracted following the same procedure as for the rest of samples. Analyses were performed with using LC-MS system from Agilent Technologies (1290 Infinity LC consisting of a degasser, two binary pumps, and a thermostated autosampler coupled with 6550 iFunnel ESI-QTOF-MS detector). Chromatographic separation was achieved with RP-column (Zorbax Extended-C18 Rapid Resolution 2.1 × 50 mm, 1.8 μm; Agilent). 0.5 μL of samples were injected into the column thermostated at 60°C. The system was operated at a flow rate of 0.6 mL/min with solvent A consisting of water and 0.1% formic acid and solvent B of acetonitrile with 0.1% formic acid. The total time of analysis was 15 minutes per sample.
Data were collected separately for positive (+) and negative (-) ion modes on a Q-TOF operated in full scan mode (*m/z* 50 to 1000) recording 1 scan per second. The drying gas flow rate was 12 L/min, the capillary voltage was 3000 V for positive and 4000 V for negative ion mode, respectively and the nozzle voltage was set to 1000 V.
Accurate mass measurements were obtained by means of an automated calibrant delivery system that continuously introduces a calibration solution containing reference masses at *m/z* 121.0509 (protonated purine) and *m/z* 922.0098 (protonated hexakis (1H, 1H, 3H-tetrafluoropropoxy) phosphazine or HP-921) in positive ion mode; and *m/z* 47 119.0363 (proton abstracted purine) and *m/z* 966.0007 (formate adduct of HP-921) in negative ion mode.

Data treatment. Raw data were cleaned of background noise and unrelated ions using Molecular Feature Extraction (MFE) algorithm through MassHunter Qualitative Analysis software (B.06.00, Agilent Technologies). The MFE created a list of all possible ions, merging co-eluting and correlating ions (different charges, adducts and neutral losses) corresponding to the same molecule into a single feature. Each feature is described by mass, retention time and abundance. The limit for the background noise was set to 1000 counts. Allowed adducts were +H, +Na, +K in positive ion mode, −H, +HCOO for negative ion mode and neutral loss of water in both ionization modes.

Alignment and quality assurance (QA) procedure were performed in Mass Profiler Professional 12.6.1 (Agilent) software. Features found across different samples were aligned with 1% tolerance for retention time and 15 ppm window for the mass. QA procedure led to the selection of features

with good repeatability. Features detected across at least 80% of QCs (for norm-carbohydrate meal group and/or for high-carbohydrate meal group) with CV <30% (as calculated for the QC samples) were kept.

Afterwards, another data filtering was performed. Metabolites present in at least 80% in at least 1 out of 10 sample groups (0′, 30′, 60′, 120′, 180′ time points for norm- and high-carbohydrate meal) were chosen. Then, dedicated filtering for each comparison was performed: metabolites present at least in 80% of samples from each group were forwarded for statistical analysis.

Based on the relation between time points and metabolite's intensity, areas under the curve (AUCs) were calculated with dedicated script in R (version 70 3.4.3, https://www.R-project.org/). The exact areas under curves for each metabolite were approximated using trapezoid rule.

Statistical analysis. Statistical analysis was performed on obtained AUCs. Patients with risk variants (homozygous CC) were compared to the patients with protective alleles (homozygous TT) in rs340874 of PROX1 gene. Norm-carbohydrate meal group was analyzed independently from high-carbohydrate meal group.

To select significant metabolites, volcano plots constructed for p(corr) and VIP (variable importance in projection) were built based on partial least square discriminant analysis (PLS-DA) models by using the SIMCA software (13.0.3 Umetrics). The acceptable values for p(corr) were > 0.5 and < -0.5, and >1 for VIP. Additionally, for each significant metabolite, *p*-value was computed in Matlab (R2012a MathWorks Inc.). The Shapiro-Wilk test was used for normality testing and then, dependently on data distribution, t-test or Mann-Whitney test were performed. Obtained *p*-values were corrected by Benjamini-Hochberg false discovery rate (FDR) test.

Identification. Only metabolites found to be significant in each comparison were identified. Identification was performed through spectral comparison of MS/MS spectra of significant compounds with the spectral data of reference compounds (HMDB, METLIN, LIPIDMAPS). Reference compounds were retrieved based on the accurate mass and isotopic distribution of the precursor and confirmed with standards.

Identified metabolites can be found in Table 2 (in the article) and Table S1 (available below). Table 2 contains metabolites and results from statistical analysis. More detailed information about compound identification (mass, retention time, fragments, and error of mass measurement) are presented in Table S1.

Pathway Analysis analysis. The Pathway Analysis was performed with MetaboAnalyst (verison 3.0) (http://www.metaboanalyst.ca/). The Pathway Analysis analyze impact of particular compounds on biochemical pathways. For this analysis 15 pathway libraries were used covering in total 1173 pathways. Significant metabolites were assigned to the pathways based on the common names, HMDB IDs, or KEGG IDs. Results from the powerful pathway enrichment analysis were combined with results from the pathway topology analysis. Next, Fisher's exact test or hypergeometric test is used. The results from the pathway analysis are presented graphically in Figure 3, and Figure S2.

Table 1. Detailed information about compounds identification[1].

Name	Molecular weight [Da]	RT [min]	Mass error [ppm]	Fragments
LysoPC 20:4 sn-2	543.3325	5.35	3	N: 303.233, 259.243, 242.080, 224.069, 168.043, 78.958
LysoPC 20:4 sn-1	543.3325	5.35	3	N: 303.233, 259.243, 242.080, 224.069, 168.043, 78.958
HDoHE	344.2351	5.70	3	N: 325.215, 299.232, 281.225, 245.156, 201.162, 173.131, 147.116, 135.116, 133.103, 121.102, 119.084, 111.081, 107.087, 97.066, 59.013, 57.032, 43.016
Ornithine	132.0899	0.25	5	N: 114.020, 85.0655, 86.993, 88.039, 44.997, 36.964
HETE	320.2351	5.70	3	N:167.108, 149.096, 59.014
HETE	320.2351	5.70	3	N:167.108, 149.096, 59.014
Linoleic acid	280.2402	7.05	3	Do potwierdzenia starndard
Leukotriene A4	318.2200	5.45	3	N: 299.198, 273.219, 255.212, 201.160, 127.075, 59.012, 44.999
Leukotriene A4	318.2200	5.45	3	N: 239.1619, 195.1737
Leukotriene A4	318.2200	5.45	3	N: 327.232, 283.242, 214.047, 196.038, 140.011, 78.959
Tetradecanedioic acid	258.1830	4.35	3	N: 279.231
LysoPE 22:6 sn-2	525.2860	5.35	1	P: 102.091, 88.075, 57.070, 43.053
LysoPE 22:6 sn-1	525.2860	5.35	1	P: 102.091, 88.075, 57.070, 43.053
HODE	298.2510	5.85	3	do potwierdzenia standard
Dodecanamide	199.1940	5.20	3	P: 502.330, 443.255, 337.274, 258.110, 184.074, 104.107, 86.096, 60.080
Taurocholic acid	515.2920	2.30	4	P: 184.073, 124.999, 104.107, 86.096
LysoPC 18:2 sn-2	519.3330	5.40	1	P: 184.073, 124.999, 104.106, 86.096, 60.080
LysoPC O-18:1	507.3690	5.95	0	P: 554.360, 184.074, 104.106
LysoPC O-16:0	481.3530	5.80	1	P: 554.360, 184.074, 104.106
LysoPC 22:4	571.3640	5.85	0	P: 500.316, 184.074, 125.000, 104.107, 86.096
LysoPC 18:3	517.3170	5.05	1	P: 145.049, 85.028, 60.080
Deoxycholic acid glycine conjugate	449.3140	4.3	1	P: 145.049, 85.028, 60.080
Acetylcarnitine	203.1160	0.25	1	P: 184.073, 86.096
PC 36:5	779.5470	7.95	0	P: 263.237, 245.226, 175.148, 109.101, 97.101, 95.085, 83.085, 81.069, 69.069, 67.054, 57.069
C18:2 Sphingosine	297.2670	5.85	0	
Linoleamide	279.2560	5.30	0	P: 526.332, 184.074, 125.000, 104.107, 86.096, 60.080
LysoPC 20:4	543.3330	5.40	0	P: 623.504
LysoPC 22:6	567.3330	5.40	1	P: 759.521, 635.5170, 184.074, 146.981, 86.096
PE 38:6	763.5150	9.40	8	
PC O-18:0/20:4	795.6140	10.20	4	

[1]Identification was performed for metabolites found to be significant in each comparison. Identification was achieved through the study of accurate mass and isotopic distribution of the precursor and product ions, comparison of obtained MS/MS spectra with the spectral data of reference compounds (CEU Mass Mediator, HMDB, METLIN, LIPIDMAPS) and confirmation with standards. P: positive ion mode; N: negative ion mode.

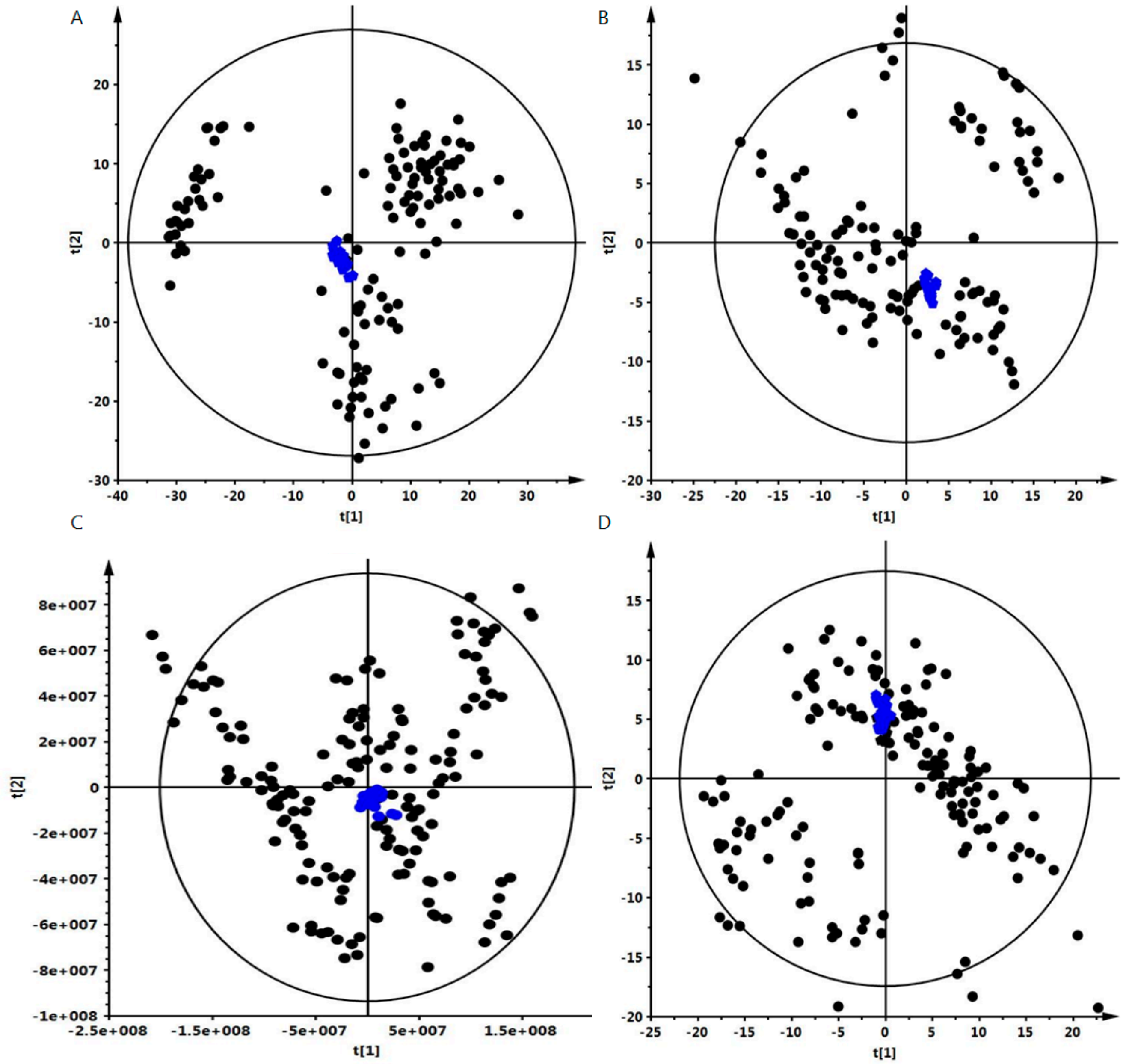

Figure S1. The QC of performed analyses - PCA plots with marked QC samples (blue color). Upper plots present marked QC samples for the NC-meal ESI + (panel A) and ESI- (panel B), for the HC-meal ESI+ (panel C) and ESI- (panel D).

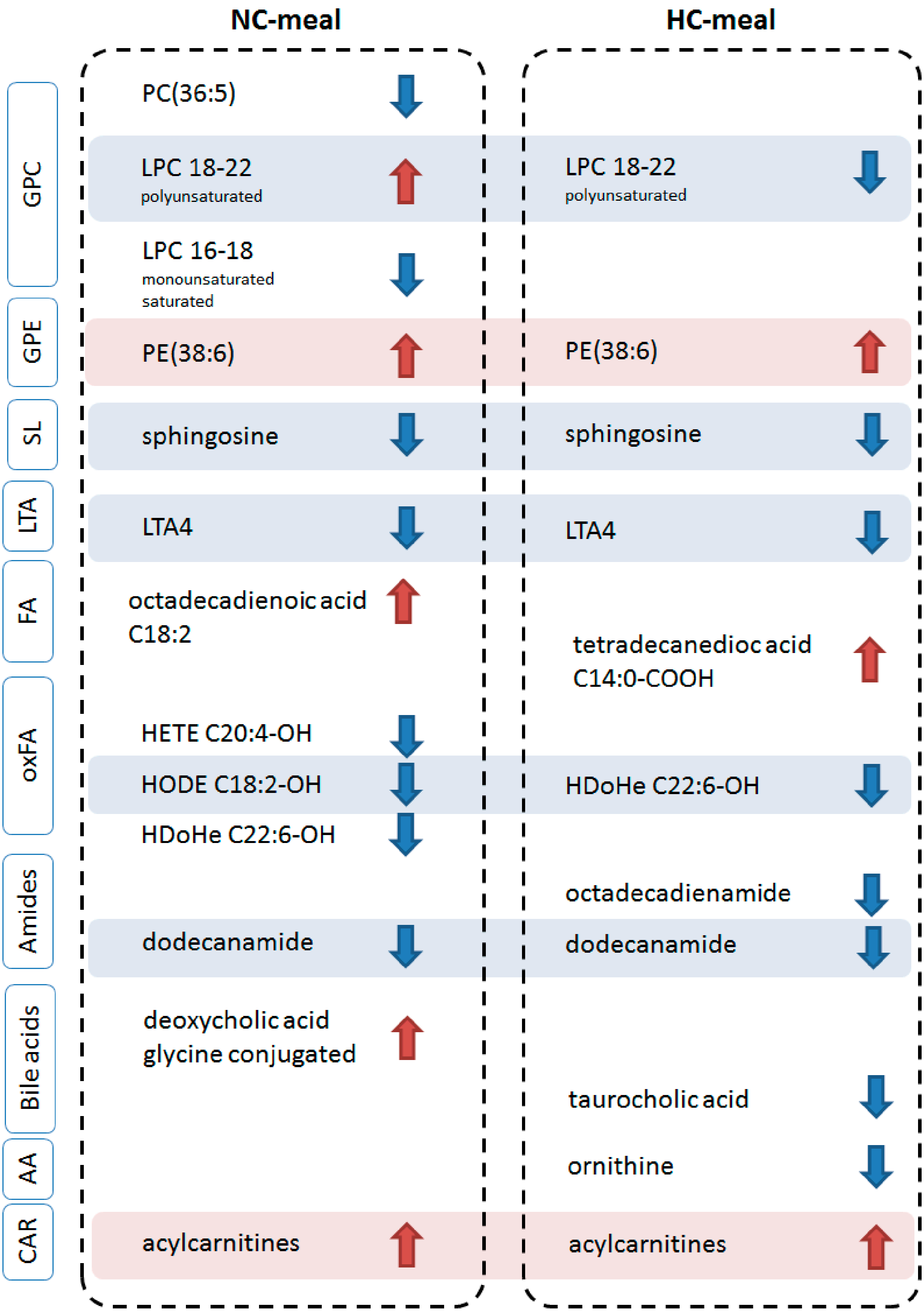

Figure S2. The summary of metabolic alterations observed after NC- and HC-meal intake. GPC: Glycerophosphocholine; GPE: Glycerophosphoethanolamine; SL: Sphingolipid; LTA: Leukotriene; FA: fatty acid; oxFA: oxidized fatty acid; AA: amino acid; CAR: carnitine. Blue arrows indicate a decrease of the concentration of metabolite in CT/TT genotype in comparison to the CC genotype. Red arrows indicate an increase of the concentration of metabolite in CT/TT genotype in comparison to the CC genotype. Transverse stripes show metabolites common between two meals.
